# Metal-free carbocatalyst for room temperature acceptorless dehydrogenation of N-heterocycles

**DOI:** 10.1126/sciadv.abl9478

**Published:** 2022-01-28

**Authors:** Haitao Hu, Yunqing Nie, Yuewen Tao, Wenyu Huang, Long Qi, Renfeng Nie

**Affiliations:** 1 School of Chemical Engineering, Zhengzhou University, Zhengzhou 450001, China.; 2 School of Chemistry and Chemical Engineering, Hubei University, Wuhan 430062, China.; 3 U.S. DOE Ames Laboratory, Ames, IA 50011, USA.; 4 Department of Chemistry, Iowa State University, Ames, IA 50011, USA.

## Abstract

Catalytic dehydrogenation enables reversible hydrogen storage in liquid organics as a critical technology to achieve carbon neutrality. However, oxidant or base-free catalytic dehydrogenation at mild temperatures remains a challenge. Here, we demonstrate a metal-free carbocatalyst, nitrogen-assembly carbons (NCs), for acceptorless dehydrogenation of N-heterocycles even at ambient temperature, showing greater activity than transition metal–based catalysts. Mechanistic studies indicate that the observed catalytic activity of NCs is because of the unique closely placed graphitic nitrogens (CGNs), formed by the assembly of precursors during the carbonization process. The CGN site catalyzes the activation of C─H bonds in N-heterocycles to form labile C─H bonds on catalyst surface. The subsequent facile recombination of this surface hydrogen to desorb H_2_ allows the NCs to work without any H-acceptor. With reverse transfer hydrogenation of various N-heterocycles demonstrated in this work, these NC catalysts, without precious metals, exhibit great potential for completing the cycle of hydrogen storage.

## Teaser

Metal-free catalysts enable room temperature release of hydrogen gas from liquid organic hydrogen storage molecules.

## INTRODUCTION

**Fig. 1.**
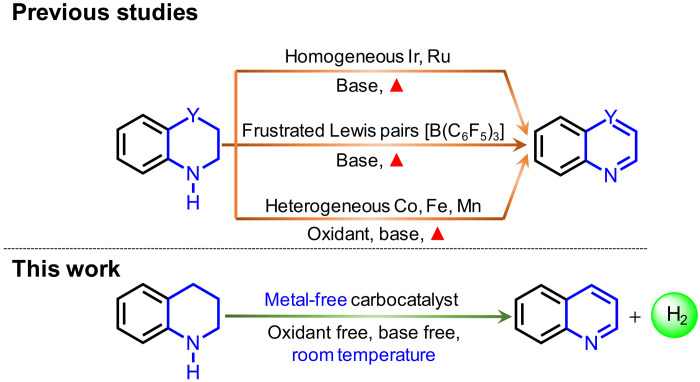
Schematic representation of the reported strategies (*8*–*14*, *16*) and our approach for catalytic dehydrogenation to release dihydrogen.

Catalytic dehydrogenation is a key transformation in the chemical and energy industry (*1*–*3*). In particular, catalytic dehydrogenation allows reversible hydrogen storage using liquid organic hydrogen carriers (LOHCs) as one of the critical technologies to achieve carbon neutrality in three decades (*4*–*7*). Effective dehydrogenation methods have been developed based on transition metal–based catalysts (Fig. 1) (*8*–*14*). However, these catalysts often rely on critical platinum group metals (*15*) with limited natural abundance and commonly suffer from the drawbacks like coking, restricting continuous large-scale application. Besides, stoichiometric acceptors such as oxidants or inorganic bases are often adopted as necessary promoters but lead to the formation of substantial side products rather than dihydrogen. Hence, effective metal-free catalyst could be highly appealing, although such catalysts are less developed, especially under acceptor-free conditions to fully release molecular hydrogen from LOHCs.

Among the few existing metal-free systems for acceptorless catalytic dehydrogenations, homogeneous frustrated Lewis pairs (FLPs) are often reported (Fig. 1) (*16*); however, most FLP systems face deactivation issues in the presence of protic reagents or moisture in air (*17*, *18*). Recently, heterogeneous metal-free carbocatalysts (e.g., carbon nanotubes, graphene, and B- or N-doped carbons) have been used in oxidative dehydrogenation reactions of ethylbenzene, alcohols, and light alkanes (*19*–*24*). The catalytic activities are normally attributed to dopants introduced with various ex situ or in situ processes (*25*). For example, oxidized carbon materials have been investigated for gas-phase oxidative dehydrogenation of propane, and surface oxygen-containing functional groups are proposed as active centers (*26*). The introduction of nitrogen can also yield carbocatalysts with active catalytic sites for the aerobic dehydrogenation (*27*–*29*), while the structures of active sites are still unclear. However, to our best knowledge, most of previous metal-free dehydrogenation of N-heterocycles required either high temperature, or excess base for hydrogen activation, or oxidants as hydrogen acceptor (*14*, *24*, *30*). Therefore, the development of an effective, robust metal-free catalyst operating at environmentally benign conditions is highly valuable but challenging.

Here, we report graphitic N-assembly carbocatalysts (NCs) for acceptorless dehydrogenation of hydroquinolines, hydroisoquinolines, or indolines to the corresponding aromatic N-heterocycles at room temperature (Fig. 1). This catalyst is also versatile for the transfer hydrogenation of aromatic N-heterocycles to corresponding saturated species as the means to close the loop of reversible hydrogen storage. The experimental results and density functional theory (DFT) calculations indicate that the observed catalytic activities result from a cooperative mechanism of closely placed graphitic nitrogens (CGNs). The CGN sites are responsible for the activation of N─H and C─H bonds in 1,2,3,4-tetrahydroquinoline (THQ) and result in the formation of labile C─H bonds adjacent to CGN sites, which leads to facile elimination of H_2_. The findings in this work pave the way for the design and development of robust metal-free dehydrogenation catalysts, critical for reversible hydrogen storage.

## RESULTS

### Synthesis and characterization

**Fig. 2.**
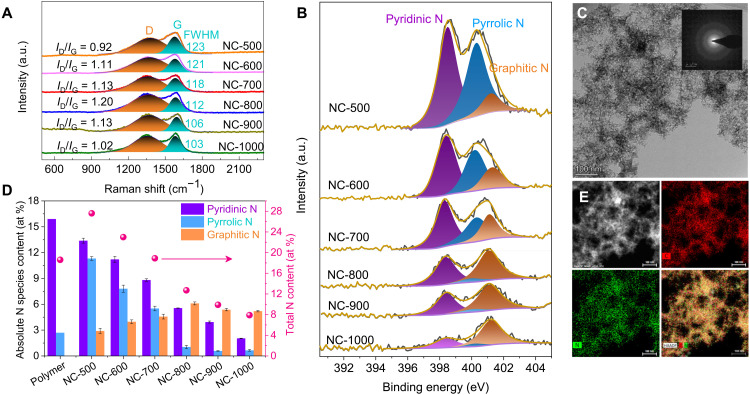
Characterization of NC catalysts. (**A**) Raman spectra, (**B**) N1s XPS spectra, (**C**) TEM image of NC-800 (SEAD image shown as the inset), (**D**) the absolute total N content and the absolute content of different N species in NCs, and (**E**) STEM elemental mapping of NC-800. a.u., arbitrary units; HAADF, high-angle annular dark-field.

NCs were synthesized via cross-polymerization of ethylenediamine (EDA) and carbon tetrachloride (CTC) using nanosilica as a hard template (*27*). After carbonization at selected temperatures between 500° and 1000°C, the template was etched, rendering six materials named as NC-*x* (*x* is the carbonization temperature). Raman spectra of NC catalysts (Fig. 2A) show the decreased width at half maximum of G band at higher carbonization temperature, ascribing to enhanced graphitization of carbon skeleton. The changes in degree of graphitization are also confirmed with wide-angle x-ray diffraction (XRD; fig. S1) patterns and electron energy loss spectra (EELS; fig. S2) of NCs, showing increased C(002) diffraction and sharp C and N K-edge peaks at higher carbonization temperature, respectively (*31*). The amorphous structure of the NCs is evidenced by transmission electron microscopy (TEM) in fig. S3 and selected area electron diffraction (SEAD; inset in Fig. 2C). All NCs displayed a type III physisorption isotherm of N_2_ (fig. S4), evidencing their mesoporous topology. The surface areas (311 to 386 m^2^/g) and pore sizes (13 to 19 nm) do not vary notably with carbonization temperatures (table S1).

The elemental composition of the NCs is analyzed with x-ray photoelectron spectroscopy (XPS), and the total nitrogen content of NCs drops from 27.6 to 7.9 atomic (at) % with rising carbonization temperatures (fig. S5A and table S2). The N extrusion is also confirmed by the downshifting and narrowing of C1s peaks of NCs as shown in fig. S5B (*32*). Analysis with both wide XPS survey and elemental analysis showed undetectable metal species (fig. S5B), eliminating the suspected contribution of impurities on catalytic activity. Elemental mapping of NC-800 determined from scanning transmission electron microscopy (STEM) indicates a good spatial overlap of C and N elements (Fig. 2E), demonstrating a homogeneous distribution of nitrogen species over the porous carbon matrix, as evidenced by diffuse reflectance infrared Fourier transform spectroscopy (DRIFTS) study (fig. S6). High-resolution N1s XPS studies (Fig. 2B) resolve pyridinic, pyrrolic, and graphitic nitrogen species in NCs, and deconvolution of the relative distribution can be obtained. With increased carbonization temperature from 500° to 1000°C, the relative graphitic nitrogen content among all nitrogen species increases significantly from 10.5 to 65.9% (fig. S7 and table S3). By combing the total nitrogen content and relative graphitic nitrogen distribution (Fig. 2D), the trend of absolute graphitic nitrogen content follows a volcanic profile, the maximum of which reaches 6.1 at % among all surface species in NC-800.

### Catalytic studies

**Fig. 3.**
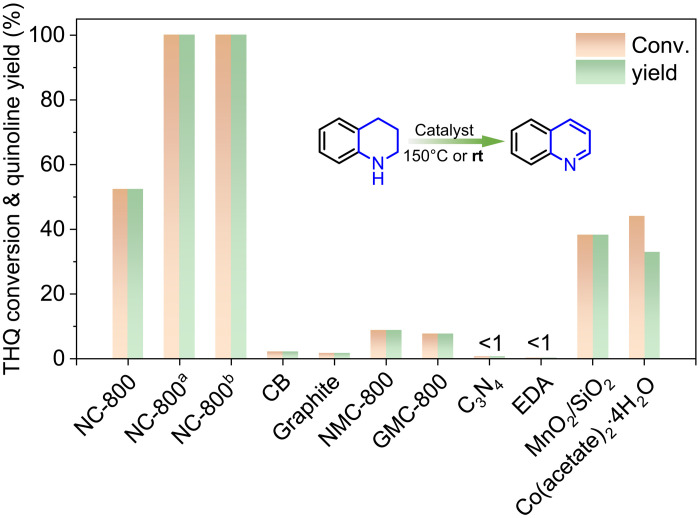
Various control experiments to obtain insights into metal-free dehydrogenation of THQ. Reaction conditions: THQ (12.5 mmol/liter), mesitylene (8 ml), catalyst (20 mg), 150°C, 1 hour, and N_2_ (1 bar). ^a^150°C, 4 hours. ^b^THQ (1.25 mmol/liter), room temperature (rt), 5 days.

The acceptorless dehydrogenation of N-heterocycles can be achieved with NC catalysts, leading to the release of H_2_. By elimination of the use of base additives or oxidants, NC catalysts can further enable applications in hydrogen storage systems and fuel cells (*33*). Within this context, we first evaluate NC materials for the metal-free dehydrogenation of 1,2,3,4-tetrahydroquinoline (THQ) under base-free and acceptorless conditions. Different solvents were initially evaluated using NC-800 at 150°C (fig. S9) under 1 bar N_2_. Mesitylene was found to be most optimal, affording a 52.4% yield of quinoline in just 1 hour (Fig. 3). Then, all six NC catalysts were tested at the same reaction condition; among all NCs, NC-800 shows the highest yield, while no product was observed in absence of catalysts. Detailed comparison of different NC catalysts will be described in a later session.

The superior performance of NC-800 is better demonstrated when benchmarked with other commonly used dehydrogenation catalysts. Under the same reaction conditions, NC-800 outperforms all metal-free materials, and 100% quinoline selectivity was observed for most catalysts, such as commercial carbon black (2.3%), graphite (1.8%), N-doped mesoporous carbon (NMC-800; 8.9%), glucose-derived mesoporous carbon (GMC-800; 7.7%), and carbon nitride (C_3_N_4_; 0.8%), with conversions shown in the parenthesis. In addition, homogeneous Co(acetate)_2_ and heterogeneous MnO_2_/SiO_2_ were examined as representative metal catalysts (*34*, *35*); compared to NC-800, both show inferior dehydrogenation activities.

With NC-800, >99% yield of quinoline can be obtained in 4 hours at 150°C [Fig. 3, fig. S10, and Table 1 (entry 1)]. In special cases with 50-fold increased concentration or solventless dehydrogenation (fig. S11), NC-800 can achieve 99.8 and 84.2% quinoline yield in 144 and 24 hours, respectively. Since our NC-800 catalyst is metal free, the amount of catalyst used in a reactor can be increased appropriately to accelerate reactions without the concern of precious metals. Note that quantitative yield of quinoline can be achieved even at room temperature with prolonged reaction time (fig. S12), together with equimolar amount of hydrogen gas (figs. S13 and S14). These results strongly demonstrate the high activity of the NC-800 catalyst for potential industrial applications over a wide window of operation temperature. To our best knowledge, dehydrogenation catalysts operating under such mild reaction conditions are very rare among literature examples even for transition metals (summarized in table S4) (*9*, *11*, *13*, *23*, *34*–*39*).

**Table 1.** Metal-free dehydrogenation of various N-heterocycles over NC-800 at room temperature and 150°C.

**Entry**	**Substrate**	**Product**	Room temperature*		**150°C^†^**	
			**Time (days)**	**Yield (%)**	**Time (hours)**	**Yield (%)**
1	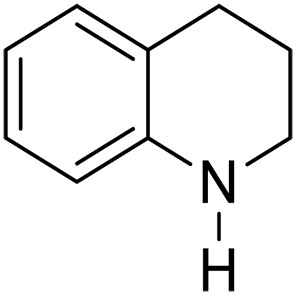	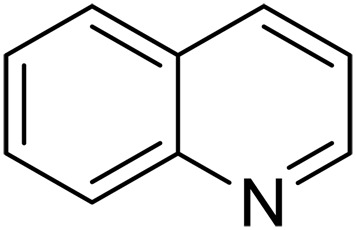	5	>99	4	>99
2	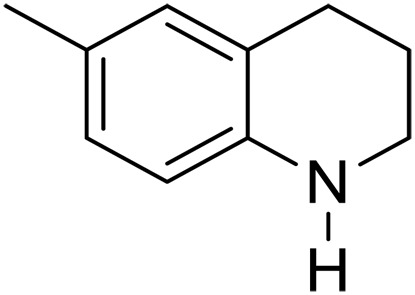	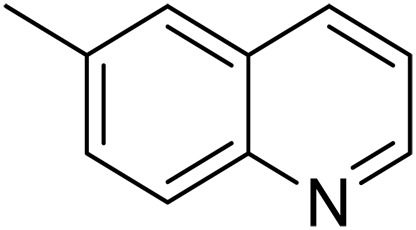	5	>99	3	>99
3	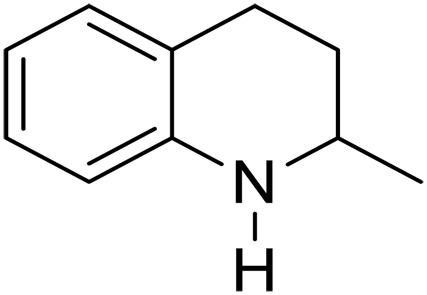	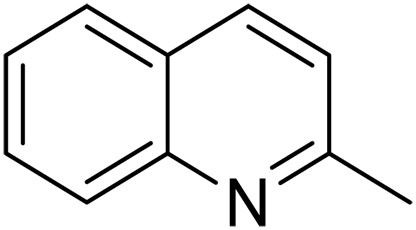	20	70	3	>99
4	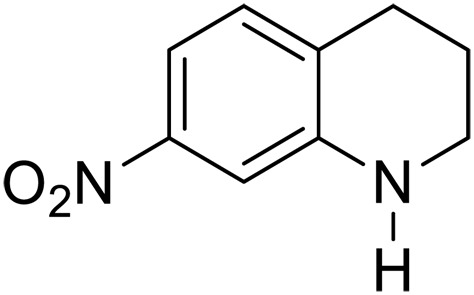	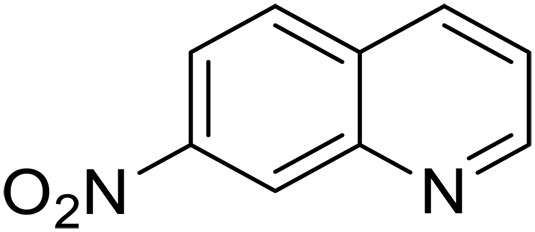	17^‡^	61	24	>99
5	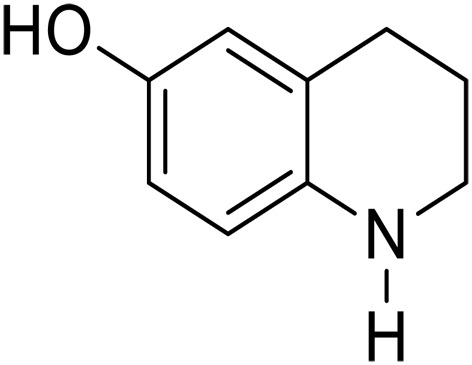	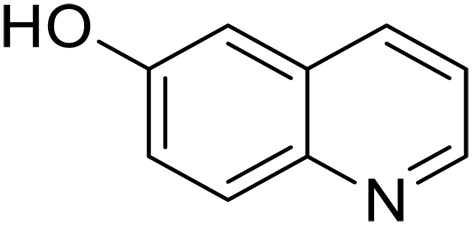	3	>99	3	>99
6	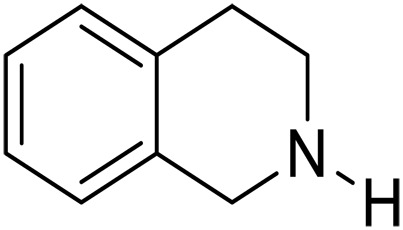	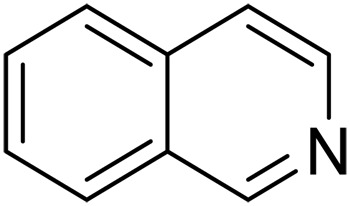	3	>99	3	>99
7	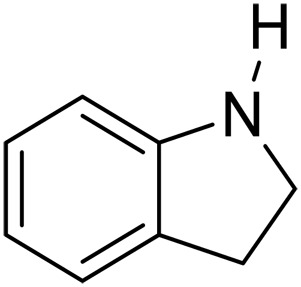	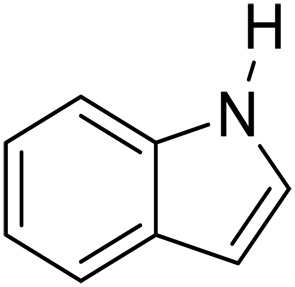	3	>99	3	>99
8	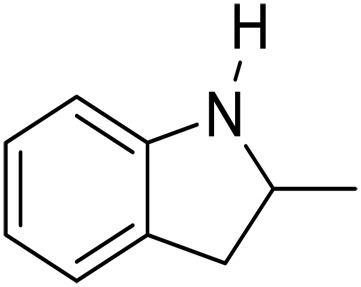	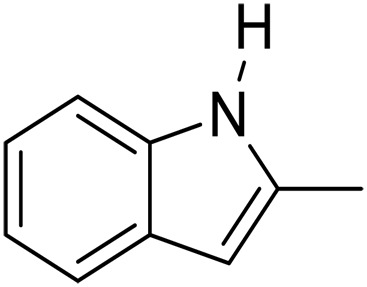	3	>99	3	>99
9	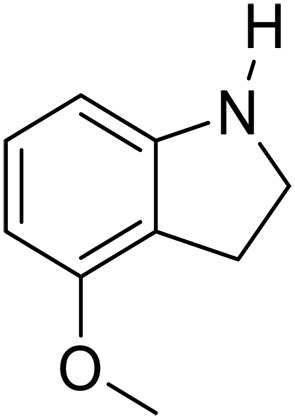	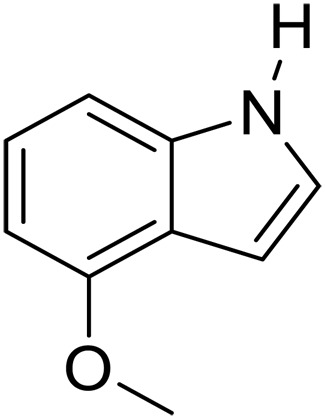	3	>99	3	>99

*Reaction conditions: substrate (1.25 mmol/liter), mesitylene (8 ml), NC-800 (20 mg), room temperature, and N_2_ (1 bar).

†Substrate (12.5 mmol/liter).

‡Substrate (0.625 mmol/liter) and NC-800 (40 mg).

We further investigated the scope of substrates at room temperature. Similar to THQ, CH_3_ or OH-substituted THQs at the sixth position were transformed to corresponding quinolines with >99% yield (entries 2 and 5, Table 1). 7-Nitro and 2-methyl–substituted THQs retard the transformation, and the corresponding substrates were converted with yields of 61 and 70%, respectively, even with longer reaction time (entries 3 and 4, Table 1), ascribing to the steric and electronic effects of substituent toward N─H bond (*36*). Besides, the NC-800 was also capable of dehydrogenating isoTHQ, indoline, and substituted indolines to afford the corresponding N-heterocycles in >99% yields (entries 6 to 9, Table 1). Upon raising the temperature to 150°C, all nine substrates can be fully converted with 100% selectivity in 4 to 24 hours (Table 1), confirming the broad applicability of NC-800 catalyst.

Catalysts able to complete the cycle of hydrogen capture and release are becoming increasingly attractive, while most reported examples are molecular catalysts based on transition metals (*8*–*14*, *40*). Therefore, after achieving the excellent performance of the NC-800 in acceptorless dehydrogenation of N-heterocycles, we proceeded to examine NC-800 for transfer hydrogenation of aromatic N-heterocycles (quinolines and indoles). The NC-800 catalyst resulted in the near-quantitative formation of 1-formyl THQs by reacting quinoline with formic acid as the hydrogen source in 23 hours at 180°C under 10 bars N_2_ (table S5). Notably, even Cl-substituted quinoline was converted to the corresponding THQ in excellent yields without any sign of dichlorination. Under the optimized conditions, isoquinoline and indole were successfully transformed in yields of >99% as well.

The stability and recyclability of the NC-800 catalyst were investigated with THQ as the substrate at 150°C. After each cycle (3 hours), the NC-800 catalyst was recovered with yield and selectivity quantified. High selectivity (>99%) was retained even after eight cycles (fig. S15). Although an activity decay was observed only after the initial run, the spent catalyst was mostly stable in the following five recycles. In the eighth run, the catalyst can achieve comparable quinoline yield (~95%) as the fresh catalyst by prolonging reaction time to 12 hours. Characterizations of spent NC-800 exhibited no apparent difference in both morphology and structure by characterization with Raman spectroscopy, XRD, XPS, and DRIFTS (figs. S6 and S16). So, the initial major activity decrease might be attributed to the surface sorption of solvent impurities and/or reaction species at very low content, resulting in partial blockage of the surface sites (*41*).

## DISCUSSION

### Identification of active sites

**Fig. 4.**
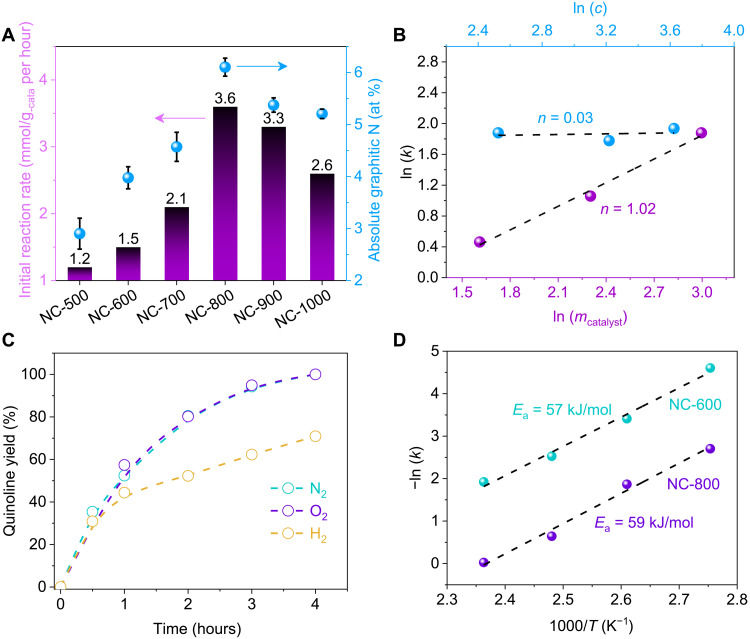
Catalytic performance for metal-free THQ dehydrogenation. (**A**) Relationship between the initial reaction rate of THQ and absolute graphitic N content within the NCs. Reaction conditions: THQ (12.5 mmol/liter), catalyst (20 mg), mesitylene (8 ml), 150°C, and 1 bar N_2_. (**B**) Measurement of rate orders for catalyst dosage and THQ concentration at early reaction stage. Reaction conditions: THQ, NC-800, mesitylene (8 ml), 150°C, and 1 bar N_2_. (**C**) Metal-free THQ dehydrogenation under N_2_, O_2_, and H_2_ atmosphere. Reaction conditions: THQ (12.5 mmol/liter), NC-800 (20 mg), mesitylene (8 ml), 150°C, and 1 bar N_2_, O_2_, or H_2_. (**D**) Calculated Arrhenius plots for NC-800 and NC-600.

To better understand the activity observed for room temperature acceptorless dehydrogenation, we further investigated the molecular origins of active sites. As previously mentioned, the reaction rate of THQ conversion is highest with NC-800 among all synthesized at different carbonization temperatures. More specifically, the initial rates are 1.2, 1.5, 2.1, 3.6, 3.3, and 2.6 mmol/g_-catal_ per hour for NC-500 to NC-1000 catalysts, respectively (fig. S17). We further plot the reaction rates against the absolute quantities of different N functionalities (from the deconvolution of the XPS spectra). A second-order polynomial relationship can be found only with graphitic N (Fig. 4A) but not with other N species (fig. S18 and table S6), indicating that the crucial role of graphitic N assembles in catalysis. Although graphitic N also exists in C_3_N_4_ and commonly studied N-doped carbon, we found that C_3_N_4_ and EDA are inactive for THQ dehydrogenation (Fig. 3). These results strongly suggest that the active sites in NCs are not isolated, but CGN sites that reported for C─O bond hydrogenolysis in our recent work (*27*). These CGN sites derive from imidazolium intermediates induced by EDA used as the N precursor (*27*).

To further confirm the role of CGN sites for THQ dehydrogenation, we measured the apparent activation energy (*E*_a_) for NC-800 and NC-600 of markedly different graphitic N content (fig. S19). On the basis of the Arrhenius plot in Fig. 4B, the apparent *E*_a_ of NC-800 and NC-600 were calculated to be 59 and 57 kJ/mol, respectively. The nearly identical apparent *E*_a_ reveals that the active sites are probably the same for different NC catalysts, while the difference in observed activities should be ascribed to the site density because of different content of graphitic N.

### Interrogation of reactive interface

**Fig. 5.**
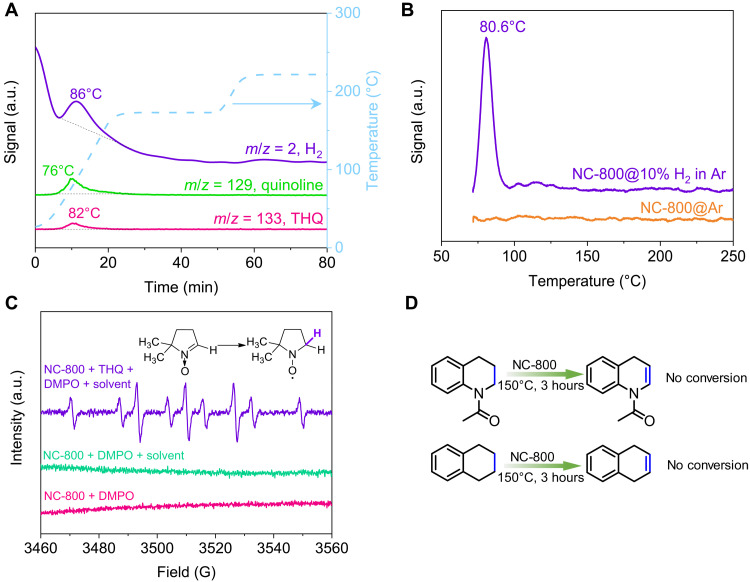
Mechanistic studies. (**A**) TG-MS study of THQ-adsorbed NC-800. (**B**) H_2_ TPD study of NC-800. (**C**) EPR analysis of reaction solutions after mixing NC-800 with DMPO, solvent, and/or THQ at 150°C. (**D**) Control experiments of dehydrogenation over NC-800. Reaction conditions: substrate (12.5 mmol/liter), NC-800 (20 mg), mesitylene (8 ml), 150°C, 3 hours, and 1 bar N_2_.

To understand the sorption reaction with NC-800, the temperature programmed desorption of THQ was studied with thermogravimetry mass spectrometry (TG-MS; Fig. 5A and fig. S20). THQ was presorbed onto NC-800 at a low surface coverage (0.03 mmol/m^2^) before transferred into the TG-MS instrument. The ramping started at 25°C under flowing Ar. The desorption signals of H_2_ and quinoline, rather than the starting THQ, become detectable at 45°C and peaked at 86° and 76°C, respectively. The THQ activation on NCs was also evidenced by the weakening of N─H and partial strengthening of C─H signals at 2965 and 2924 cm^−1^ in in situ DRIFTS experiment (fig. S21). Note that no signal of water was observed, indicating that the reaction is catalytic but not caused by sacrificial reduction of the NC-800 catalyst.

The reversible hydrogen splitting was further studied with H_2_ temperature-programmed desorption (TPD) measurement (Fig. 5B). After presorption of H_2_ at room temperature, the sample was purged with Ar to remove physisorbed H_2_ before the desorption ramping was initiated. A desorption signal for H_2_ was observed at ca. 81°C, close to the desorption temperature of quinoline observed with TG-MS, indicating that NC-800 can split H_2_ even at room temperature. Results from both TG-MS and H_2_ TPD studies clearly show that H_2_ and quinoline have low desorption temperature, and therefore, desorption of reaction products is likely not rate limiting even at mild reaction temperatures. To probe H_2_ desorption under that reaction condition, the conversion of THQ toward quinoline over NC-800 was carried out in the presence of O_2_ and H_2_ atmosphere (Fig. 4C).

The THQ dehydrogenation was not affected at all by O_2_, suggesting that the recombination of surface H atoms to yield H_2_ is reversible but not rate limiting. However, the rate of THQ dehydrogenation was not affected at the initial stage of the reaction by the H_2_ atmosphere but slows down when the conversion of THQ reached ca. 50% because of the equilibrium of THQ-quinoline conversion. Therefore, the reaction mechanism of NC catalysts is different from other oxidative dehydrogenation catalysts including boron nitride. Our previous studies show that the facile, reversible hydrogen can be achieved via CGN sites, which can localize charge densities at the carbon between two graphitic N atoms to readily form covalent C─H bonds (*27*).

To probe the reactivity of these active C─H bonds, 5,5-dimethyl-1-pyrroline *N*-oxide (DMPO), a commonly used spin trapping agent, was added to the catalytic reaction mixtures with THQ and NC-800. After the reaction at 150°C, multiple signals of the reaction mixture were clearly observed in the electron paramagnetic resonance (EPR) spectrum (Fig. 5C). These signals can only be observed for aminoxyl radical formed after DMPO binds with hydride on metal surfaces or hydrogen radicals (*42*). Since there is no EPR signal when just mixing catalyst and solvent in the absence of the THQ, the active hydrogens are most likely formed during the dehydrogenation reaction. To rule out the formation of hydrogen or other radicals, we also attempted to add other radical scavengers (e.g., phenol, 2,2,6,6-tetramethyl-1-piperidinyloxy (TEMPO), and benzoic acid) in the reaction mixtures (fig. S22) but found little impact on the dehydrogenation reaction. Therefore, the transient surface C─H bond is covalent but labile enough to be readily cleaved, which explains the observed catalytic activities without the need of acceptors under mild reaction condition (*27*).

Kinetic studies with THQ showed that the apparent rate law is 1.0-order dependence on catalyst NC-800 and zero order on THQ (Fig. 4D and fig. S23). The rate orders indicate that there is no internal mass transfer limitation and, more importantly, the adsorption of THQ is not turnover limiting. The hydrophobic carbon surface favors the sorption of aromatic compounds to further increase the surface concentration of THQ.

On the basis of all mechanistic and kinetic studies, the rate-determining step is most probably correlated with the bond cleavages in surface-bound THQ. Control experiments were performed using 1-acetyl-1,2,3,4-tetrahydroquinoline and 1,2,3,4-tetrahydronaphthalene as substrates (Fig. 5D). In either case, there is no N─H bond present, and dehydrogenation product was not detected. The results of control experiments suggest that the direct formation of C═C bond is disfavored, and involvement of the N─H bond may lower the dehydrogenation step for THQ. According to related literature on THQ dehydrogenation with both homogeneous (*43*) and heterogeneous catalysts (*9*), 3,4-dihydroquinoline, the intermediate after the elimination of the first H_2_, was observed to tautomerize to 1,2-isomer with the reformation of the N─H bond. The 1,2-isomer, rather than the 3,4-isomer, seems to undergo further dehydrogenation, implying the involvement of N─H is also necessary to the release of the second H_2_.

**Fig. 6.**
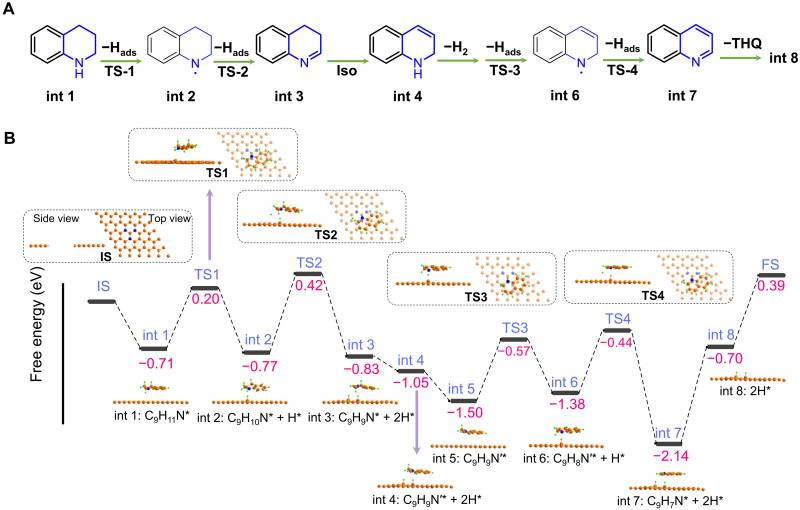
Mechanism of NC-800 catalyzed THQ dehydrogenation. (**A**) Hypothesized reaction mechanism based on experimental results and (**B**) potential-free energy diagrams with CGN as the active site by DFT calculations. The label IS represents the initial state, and the subsequent labels int 1 to int 8 represent a series of intermediate states. The labels TS1 to TS4 (TS is short for transition states) represent a series of transition states. The orange, blue, and green spheres represent the C, N, and H atoms, respectively.

Therefore, the overall reaction route can be hypothesized and depicted in Fig. 6A and fig. S24. THQ is first adsorbed on the catalyst surface [intermediate state (int) 1], followed by sequential activation of the N─H and C─H bonds by the CGN site to form intermediate 3,4-dihydrofuroquinoline (int 3). The int 3 will be tautomerized to int 4. After desorption of the first H_2_, a second dehydrogenation step takes place with formation of quinoline (int 7) and the second H_2_ to complete the catalytic cycle.

### Theoretical studies

To achieve molecular-level understanding of CGN sites in bond activation, we performed DFT calculation based on our hypothesized mechanism (Fig. 6A). According to our previous study (*27*), the periodic graphene model with ternary N cluster is simulated as one of the representative CGN sites for THQ dehydrogenation in Fig. 6B. As the first step, the N─H bond of THQ is cleaved, and H atom is transferred to a graphitic carbon bonded to two N graphitic atoms, with the formation of int 2 and surface C─H bond. This step (from int 1 to int 2) was calculated to be exothermic by 0.06 eV with a barrier of 0.91 eV. Then, the C_α_─H of THQ is activated and captured by a second graphitic carbon neighboring CGN sites to form int 3. This step (from int 2 to int 3) is exothermic by 0.06 eV with a barrier of 1.19 eV. The following tautomerization of int 3 to int 4 is exothermic by 0.22 eV, and the reformation of the N─H bond is necessary to facilitate the following dehydrogenation (*9*). After that, the first H_2_ was liberated off the catalyst surface, and the active site was restored (int 5). Next, the dehydrogenation of int 5 occurs to form the surface-bound quinoline (int 7) via int 6, like the release of the first H_2_. We calculated that the energy barriers for these two steps (int 5 to int 6 and int 6 to int 7) are 0.93 and 0.94 eV, respectively; the former step is endothermic by 0.12 eV, while the latter one is exothermic by 0.76 eV.

For better comparison, we also simulate the same reaction paths with isolated graphitic N (IGN) sites that are suggested as the active site for conventional N-doped carbon (fig. S25) (*44*). The energy barriers of the four dehydrogenation steps are 1.50, 1.46, 1.16, and 1.21 eV on the IGN site, which are higher for every dehydrogenation step compared to PGN site. Besides, on the basis of the energetic span model (*45*), the energetic span (δ*E*) of the reaction was calculated to be 2.53 and 2.94 eV on CGN and IGN sites, respectively. A lower δ*E* indicated the higher catalytic activity of the CGN site. Although the desorption of quinoline (C_9_H_7_N*) was calculated to be of the highest barrier for both CGN (1.44 eV) and IGN (1.68 eV) sites, the presence of reactant or solvent molecules may facilitate desorption of quinoline under reaction conditions. Aside from desorption energy, all calculated reaction energies are in good agreement with our experimental findings.

In conclusion, we present NCs with room temperature activities for acceptorless dehydrogenation of multiple N-heterocycles. On the basis of extensive mechanistic and kinetic studies, we show that CGNs, derived from the diamine precursors, can lead to labile C─H bonds after dehydrogenation and thus accelerate the recombination of sorbed hydrogens to form H_2_. The CGN sites are of lower energy barriers in every elemental step of THQ dehydrogenation when compared to IGN sites of traditional N-doped carbon materials. Because of the markedly different mechanism of CGN as the active sites, NCs can catalyze dehydrogenation effectively without the use of any base or oxidant as additives. Tuning carbonization temperature of NCs shows profound impact on the reaction rates but not on the apparent activation energy, suggesting that all NCs are of the same type of active sites. The optimized NC-800 shows a wide window for operation temperature with quantitative selectivity and manifested potential for high recyclability. The same NC-800 catalyst can also catalyze the transfer hydrogenation of N-heterocycles as a step forward to complete the reversible hydrogen storage, with N-heterocycles as the hydrogen carrier. This discovery unveils great potential of metal-free carbocatalysts with innovative surface sites for the catalytic transformation of N-heterocycles, critical for chemical manufacturing and energy storage.

## MATERIALS AND METHODS

### Synthesis of graphitic N-assembly carbon materials (NC-*x*)

Typically, CTC (24 g), EDA (10.8 g), and nanosilica (3.2 g) were added into a 100-ml round-bottomed flask. The mixture was stirred and refluxed at 90°C in ambient atmosphere for 6 hours. Afterward, CN polymer was obtained after solvent evaporation. The residue was carbonized (3°C min^−1^, 5 hours) at a given temperature under a flow of N_2_ (99.999%). The black powder was treated with a solution of 5 weight (wt) % HF to remove Si. The suspension was filtrated, washed with water for ≥10 times, and dried under vacuum at 100°C for 24 hours. The final sample was named as NC-*x* (*x* = carbonization temperature) and stored at a desiccator before usage.

### Synthesis of C_3_N_4_

Ten grams of melamine was heated in a crucible to 550°C at a heating rate of 2°C min^−1^ under air and kept for 4 hours at 550°C.

### Synthesis of glucose-derived mesoporous carbon (GMC-800)

Five grams of glucose was transferred into a crucible, heated to 800°C at a heating rate of 5°C min^−1^ under a flow of N_2_ (99.999%), and kept for 4 hours at 800°C.

### Synthesis of N-doped mesoporous carbon (NMC-800)

Five grams of glucose and 5 g melamine were physically mixed in mortar for 30 min. The powder was transferred into a crucible, heated to 800°C at a heating rate of 5°C min^−1^ under a flow of N_2_ (99.999%), and kept for 4 hours at 800°C.

### Synthesis of MnO_2_/SiO_2_

A total of 0.72 g of nano SiO_2_ was added into 50 ml of water containing 0.827 g of 50 wt % manganese nitrate aqueous solution and stirred at 50°C for 6 hours. Afterward, the solvent was removed by rotary evaporation. The solid was heated to 300°C under N_2_ with a rate of 2°C min^−1^ and kept at that temperature for 2 hours. The product was named as MnO_2_/SiO_2_, and the MnO_2_ loading was detected to be 27.8 wt %.

### Characterizations

Powder XRD was performed on a Bruker D8A25 diffractometer with CuKα radiation (λ = 1.54184 Å) operating at 30 kV and 25 mA. Raman spectra were collected at room temperature from 100 to 4000 cm^−1^ with a 514.5-nm argon ion laser (Renishaw Instruments). N_2_ physisorption was carried out at −196°C using the TriStar II auto-adsorption analyzer (Micromeritics). Before adsorption measurements were taken, the samples were degassed at 250°C overnight. The total pore volume was determined from the aggregation of N_2_ vapor adsorbed at a relative pressure of 0.99. The specific surface area was calculated using the Brunauer-Emmett-Teller (BET) method, and the pore size was estimated using the Barrett-Joyner-Halenda (BJH) method from the desorption branch of the isotherms. TEM images were obtained using an accelerating voltage of 200 kV on a JEOL-135 2010F TEM. STEM images were recorded using the FEI Titan G260-300 TEM operated at 200 kV. The inductively coupled plasma mass spectroscopy was measured on NexION 300X (PerkinElmer) to detect the possible metal impurities in the NCs.

X-ray photo-electron spectra (XPS) were recorded on a PerkinElmer PHI ESCA system. The binding energy values were strictly calibrated using the C1s peak at 284.6 eV. The N1s peak of assigned N1s species was fitted at an extremely narrow range for the binding energy, including 398.3 to 398.5 eV for pyridinic N, 400.0 to 400.2 eV for pyrrolic N, and 401.1 to 401.2 eV for graphitic N. The full width at half maximum values of the fitted peaks were restricted to a range of 1.4 to 1.7 eV.

The TPD was performed on AutoChem II 2920 (Micromeritics, USA) equipped with a thermal conductivity detector (TCD). In a typical test, 200 mg of catalyst was pretreated at 200°C for 1 hour in an Ar flow of 20 ml/min and then cooled down to 50°C. Then, the sample was treated with 10% H_2_/Ar (15 ml/min) for 60 min and swept with Ar (20 ml/min) for 60 min. Subsequently, the sample was heated to 600°C with the heating rate of 10°C/min under 10% H_2_/Ar (15 ml/min).

### EPR test

EPR spectra were recorded in PhCMe_3_ on a Bruker A300 spectrometer (*X* band, 9.43 GHz, amplitude = 0.7 G) at room temperature. All analyses were carried out as follows: before acquiring the spectra, combinations of catalyst NC-800 with solvent, DMPO, and/or THQ were charged into the reaction vessel. The whole system was evacuated and then flushed with N_2_ before warming up to 150°C for 2 hours. Then, the suspension cooled down to room temperature and tested for EPR.

### TG-MS test

TG-MS experiments were performed using SETSYS Evolution (Setaram) with a quadrupole mass spectrometer QMG 700 (Pfeiffer) directly coupled by a SuperSonic system (Setaram). TG-MS curves were recorded under Ar atmosphere (flow rate of 20 ml·min^−1^), the temperature was raised from 30° to 150°C and kept for 30 min, then heated to 200°C and kept for 30 min, and then heated to 500°C, with the heating rate of 10°C min^−1^. The MS signals corresponding to H_2_ [mass/charge ratio (*m*/*z*) = 2], quinoline (*m*/*z* = 129), and 1,2,3,4-tetrahydroquinoline (*m*/*z* = 133) were monitored in multiple ion detection mode.

### Catalytic dehydrogenation

Dehydrogenation of THQ was carried out in a 25-ml glass tube. In a typical operation, 8 ml of mesitylene, 0.1 mmol of THQ, and 20 mg of catalyst were added into the tube. Then, the tube was vacuumed, plugged in an N_2_ balloon, and heated to the desired temperature under stirring [600 revolutions per minute (rpm)]. After the reaction, the reactor was quickly quenched in room temperature water, and the solid catalyst was separated by centrifugation. The liquid was diluted with EtOH and analyzed by gas chromatography (GC) (Fuli 9790Plus) with a 30-m capillary column (HP-5) and a flame ionization detector (FID). All products were confirmed with GC-MS, although intermediates, including 1,2-dihydroquinoline and 3,4-dihydroquinoline, could not be detected because of instability under air. Naphthalene was used as the internal standard for product quantification using standard solutions of the substrates and products. Conversions and yields were further calculated on the basis of the calibration curves established on the dependency of the chromatographic areas as a function of concentration. The initial rates are determined, using data below 20% conversion. In the recycling study, the catalyst can be recovered by centrifugation and then washed four times with 20 ml of EtOH each, followed by vacuum drying at 80°C overnight for the next cycle.

### Catalytic transfer hydrogenation

The catalytic hydrogenation of quinoline was carried out in a 10-ml stainless steel high-pressure reactor (Teflon inner). In a typical operation, quinoline (0.1 mmol), formic acid (2.5 mmol), catalyst (20 mg), and mesitylene (5 ml) were added to the reactor. Then, the reactor was sealed, purged with N_2_ for three times, pressurized to 10 bars, and heated to 180°C by stirring (600 rpm). After the reaction, the reactor was quenched in cold water to stop the reaction quickly. The liquid was diluted with EtOH and analyzed by GC (Fuli 9790Plus) with a 30-m capillary column (HP-5) and an FID. All products were confirmed by GC-MS (Agilent 5977A MSD). Naphthalene was used as the internal standard for product quantification using standard solutions of the substrates and products.

### Simulation details

Two catalytic models were designed in our study as the follows: model 1—possible coordination structures of three graphitic N atoms anchored in perfect graphite sheet; model 2—only one graphitic N atom was anchored in perfect graphite sheet. These theoretical models have been commonly considered from our experimental results. Subsequently, one adsorbate (THQ) was placed on the active sites of the two surfaces. Last, initial adsorption systems were constructed in the same rhomboid simulation lattice built with three dimensions of *x* = 12.36 Å, *y* = 12.36 Å, and *z* = 20.04 Å.

All DFT calculations in this study were performed using the DMol (*46*) program package in Materials Studio 2018. The exchange and correlation terms were determined using the generalized gradient approximation in the form proposed by Perdew, Burke, and Ernzerhof (PBE) (*47*, *48*). Core trebarent was adopted as DFT semicore pseudopots to conduct metal relativistic effect. A double numerical plus polarization function basis set was also used. A semi-empirical van der Waals correction accounted for the dispersion was included through the use of the DFT-D2 method of Grimme (*46*). The cutoff was set as 4.5 Å, and a 0.005-ha smearing that was used for the orbital occupation was applied. The thresholds of energy, force, and displacement are 10^−5^ Hartree, 2 × 10^−3^ Hartree per atom for the maximum force, and 5 × 10^−3^ Å for displacement. Self-consistent field procedures were performed with a convergence criterion of 10^−6^ Ha on the total energy to achieve accurate electronic convergence. In addition, *k*-point mesh for sampling the Brillouin zone was also tested, and a (3 × 3 × 1) *k*-point mesh is accurate enough to acquire converged adsorption energies.

Then, the complete linear synchronous transit/quadratic synchronous transit (LST/QST) method (*49*) was used to locate transition-state structures of each primary step. All transition states are validated by vibration analyses with only one imaginary frequency. Last, the free energies of the first step dehydrogenation process of tetrahydroquinoline on the two models’ surfaces are calculated

ΔG=ΔE+ΔZPE−TΔS

Where ∆*E* is the reaction energy of a given reaction step and can be obtained from DFT calculations, and ∆ZPE and ∆*S* are the corrected zero point energy and entropy, respectively.
