# Supplementary material for: Metal-free carbocatalyst for room temperature acceptorless dehydrogenation of N-heterocycles
Source: Sci Adv. 2022 Jan 28;8(4):eabl9478. doi: 10.1126/sciadv.abl9478 (PMC8797793; doi:10.1126/sciadv.abl9478)
Supplement: Supplementary file 1 — Supplementary Materials and Methods Figs. S1 to S25 Tables S1 to S6 [file sciadv.abl9478_sm.pdf]

Science Advances
AAAS

Supplementary Materials for

Metal-free carbocatalyst for room temperature acceptorless dehydrogenation of N-heterocycles

Haitao Hu, Yunqing Nie, Yuewen Tao, Wenyu Huang, Long Qi*, Renfeng Nie*

*Corresponding author. Email: rnie@zzu.edu.cn (R.N.); lqi@iastate.edu (L.Q.)

Published 28 January 2022, *Sci. Adv.* **8**, eabl9478 (2022)
DOI: 10.1126/sciadv.abl9478

This PDF file includes:

Supplementary Materials and Methods
Figs. S1 to S25
Tables S1 to S6

Materials and Methods

Materials. Ethylenediamine (≥99.5%), carbon tetrachloride (≥99.5%), melamine (CP), glucose (≥99.5%), 1,2,3,4-tetrahydroquinoline (THQ, ≥98%), 6-methyl-1,2,3,4-tetrahydroquinoline (≥97%), 2-methyl-1,2,3,4-tetrahydroquinoline (≥98%), 7-nitro-1,2,3,4-tetrahydroquinoline (≥97%), 6-hydroxy-1,2,3,4-tetrahydroquinoline (≥98%), tetrahydroisoquinoline (isoTHQ, ≥98%), indoline (≥98%), 2-methyl-indoline (≥97%), 5-methoxy-indoline (≥97%), quinoline (≥98%), 2-methyl-quinoline (≥98%), 4-methyl-quinoline (≥98%), 8-methyl-quinoline (≥98%), 6-methoxy-quinoline (≥96%), 8-chloroquinoline (≥98%), isoquinoline (≥97%), indole (≥99%), 1-acetyl-1,2,3,4-tetrahydroquinoline (98%), 1,2,3,4-tetrahydronaphthalene (97%), $CDCl_3$-*d* (99.5%, 99.9 atom% D) were purchased from Aladdin Chemical. Mesitylene (99%), dimethyl sulfoxide (99%), benzonitrile (99%), dimethylformamide (99%), ethanol (AR), decalin (AR) were purchased from Sinopharm Chemical. 5,5-Dimethyl-1-pyrroline N-oxide (DMPO, 98%), tetramethylpiperidine oxide (TEMPO, 98%) were purchased from Macklin Chemical. Formic acid (98%), phenol (AR), benzoic acid (99%), hydrofluoric acid (AR), were purchased from Energy Chemical. 50 wt% manganese nitrate aqueous solution, cobalt acetate tetrahydrate (≥99%) were purchased from Sinopharm Chemical. Nano-silica (NANOCRYL A 200, Ø 20 nm) was purchased from Evonik Degussa. Carbon black (black pearls 2000) was purchased from Cabot Corporation. H_2 (99.995%) and N_2 (99.999%) were purchased from Gas Company of Wuhan Iron & Steel Group. All chemicals were used as received.

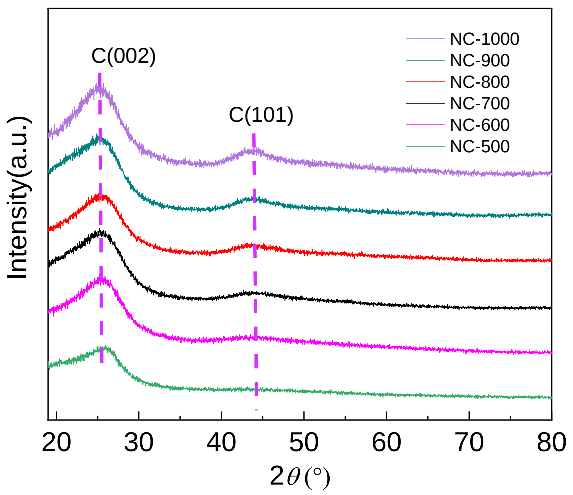

Fig. S1. XRD patterns of NCs. Only the C (002) and C (101) diffraction peaks are observed for NCs, and two of them strengthen as increasing carbonization temperature.

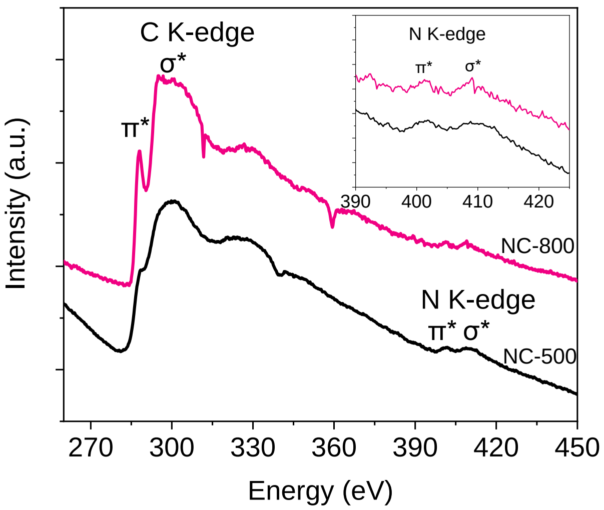

Fig. S2. Electron energy loss spectra (EELS) of NC-500 and NC-800.

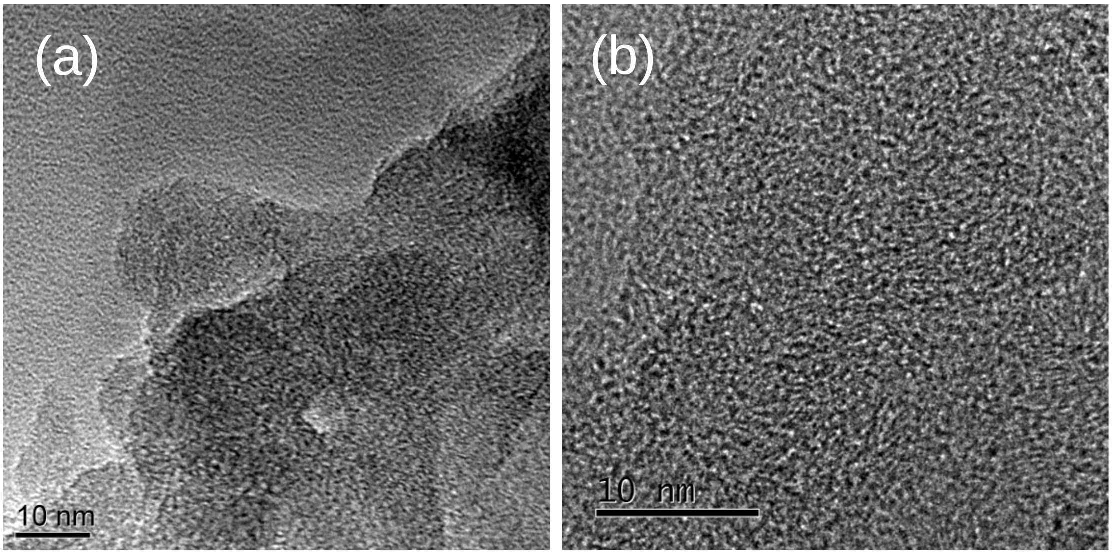

Fig. S3. HRTEM images of NC-800. The TEM images (a and b) show that the NC-800 is full of mesopores and exhibits low crystallinity over a large domain.

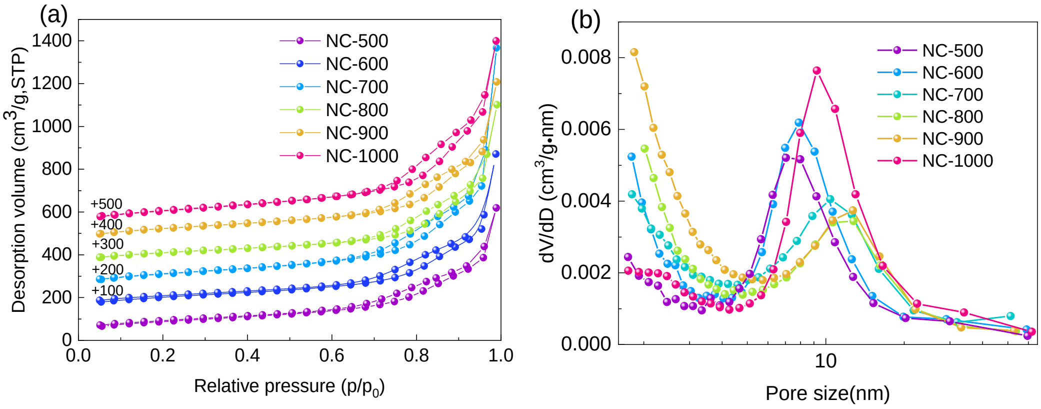

Fig. S4. Characterization of pore structure of NCs. (a) N_2 sorption isotherms and (b) pore size distributions.

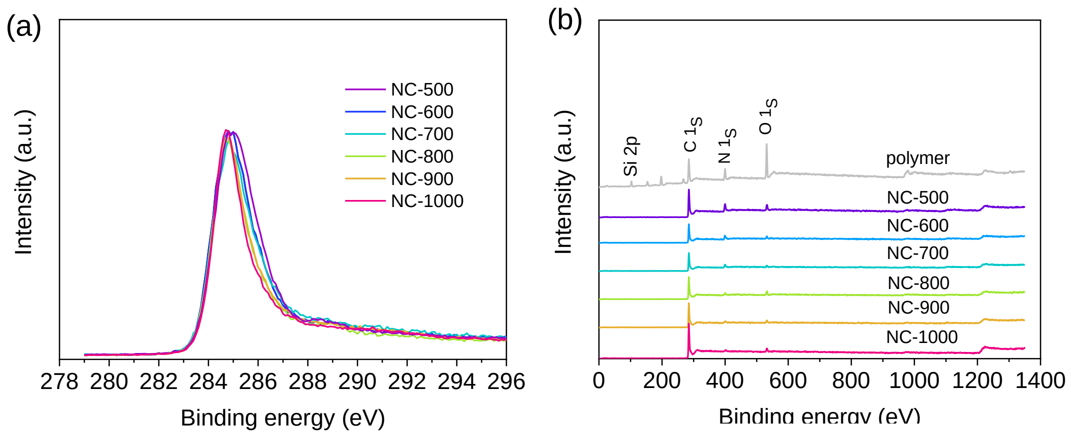

Fig. S5. XPS spectra of NCs. (a) C1s spectra and (b) wide survey spectra. The narrowing C1s peaks reveal the decreasing content of heteroatoms at high carbonization temperature. No metal impurities except C, N and O signals can be observed from wide survey spectra.

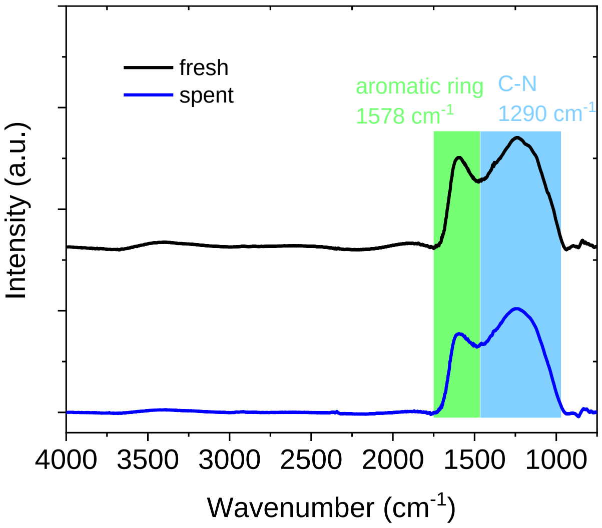

Fig. S6. The DRIFTS spectra of fresh and spent NC-800. The sample was diluted with KBr (50x) and tested under flowing He at 25 °C, only graphitic *sp*^{2} domains (1578-1593 cm^{-1}) and C−N bond (1269-1290 cm^{-1}) can be observed.

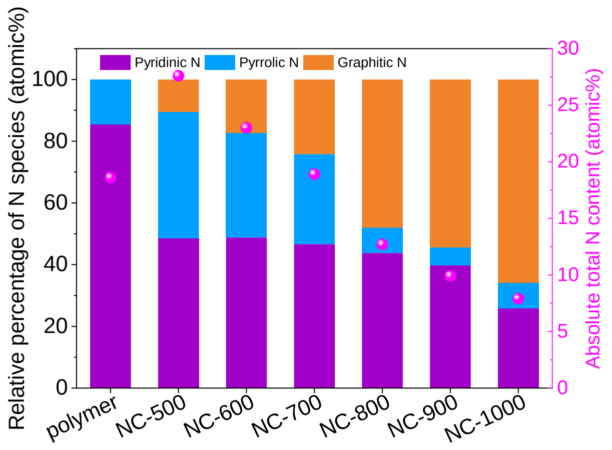

Fig. S7. The relative percentage of different N species in NCs. Higher carbonization temperature results in the decrease of both pyridinic and pyrrolic N, while graphitic N increases with temperature at 500-1000 °C.

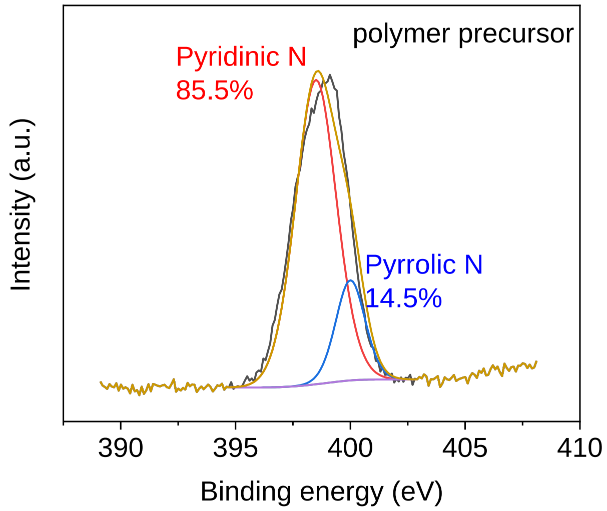

Fig. S8. N1s XPS spectrum of NC polymer precursor.

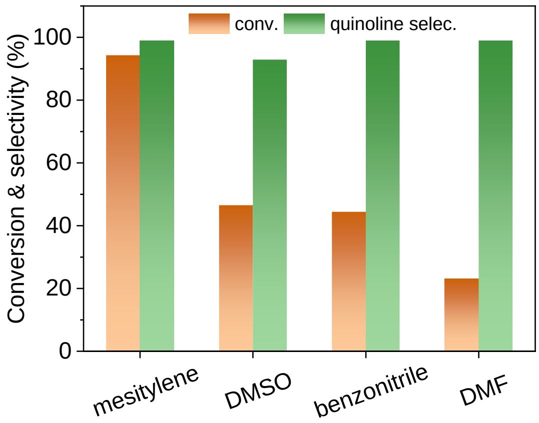

Fig. S9. Influence of solvent on THQ dehydrogenation over NC-800. Reaction conditions: THQ (12.5 mmol/L), solvent (8mL), NC-800 (20 mg), N_2 (1bar), 150 °C, 3 h.

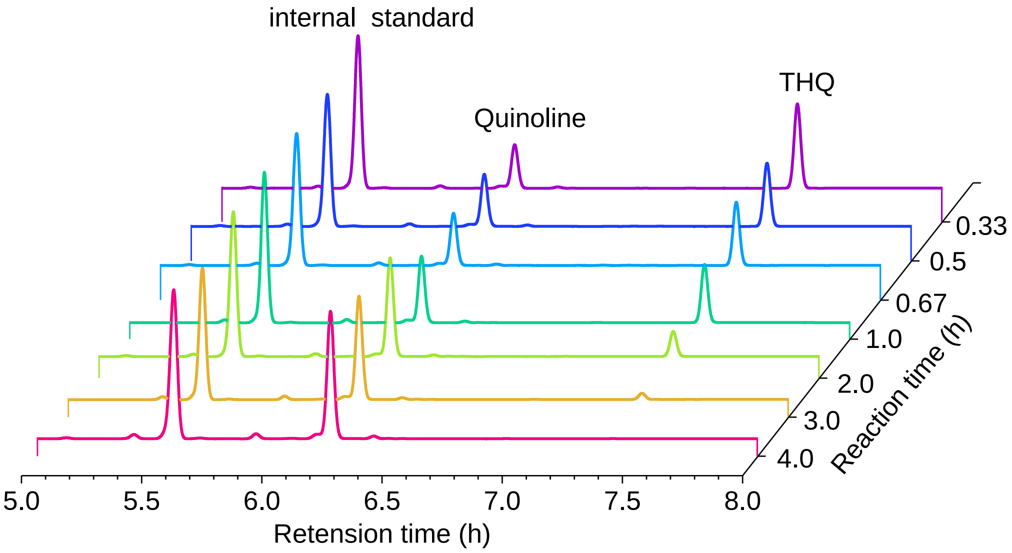

Fig. S10. GC chromatograms of the liquid products obtained from the dehydrogenation of THQ over NC-800 at different times. Reaction conditions: THQ (12.5 mmol/L), mesitylene (8 mL), NC-800 (20 mg), N_2 (1bar), 150 °C. GC temperature program: 2 min at 100 °C, 10 °C/min to 220 °C.

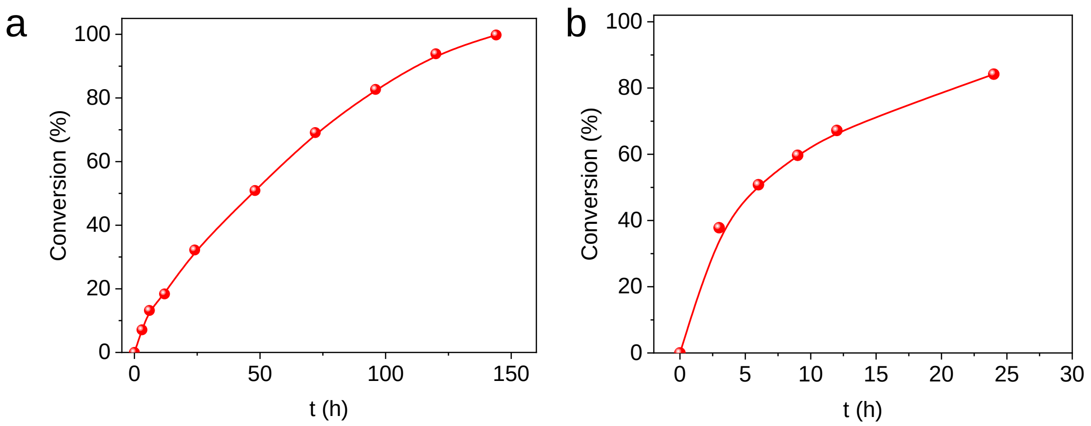

Fig. S11. **THQ dehydrogenation with increased initial THQ concentrations.** (a) Time-conversion profiles of THQ dehydrogenation over NC-800 with 50-fold increased THQ concentration. Reaction conditions: THQ (0.625 mol/L), mesitylene (8 mL), NC-800 (20 mg), 150 °C, N_2 (1 bar). (b) Time-conversion profiles of solventless THQ dehydrogenation over NC-800. Reaction conditions: THQ (1 mmol), NC-800 (20 mg), 150 °C, N_2 (1 bar).

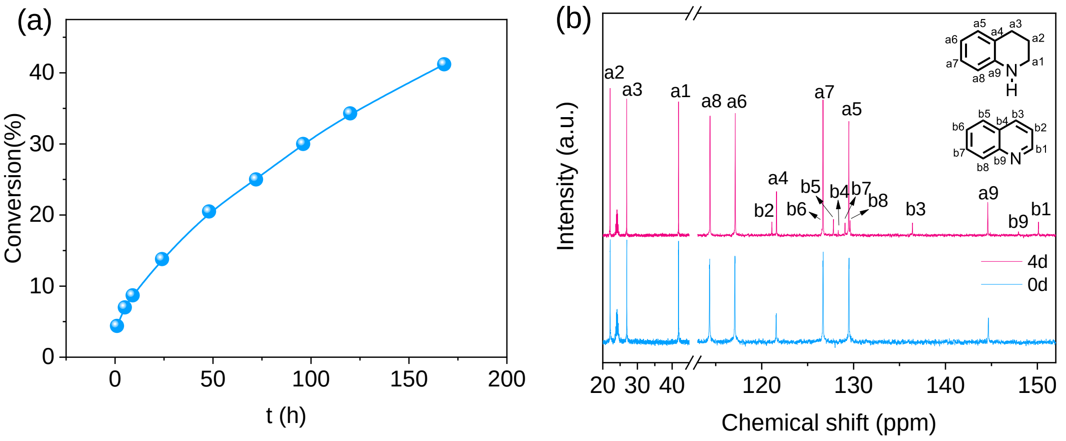

Fig. S12. Room-temperature dehydrogenation of THQ over NC-800. (a) Time profile at room temperature. Reaction conditions: THQ (12.5 mmol/L), mesitylene (8 mL), NC-800 (20 mg), room temperature, N_2 (1 bar). (b) ^{13}C-NMR spectra of dehydrogenation of THQ over NC-800. Reaction conditions: THQ (50.0 mmol/L), deuterated chloroform (2 mL), NC-800 (20 mg), room temperature, N_2 (1 bar).

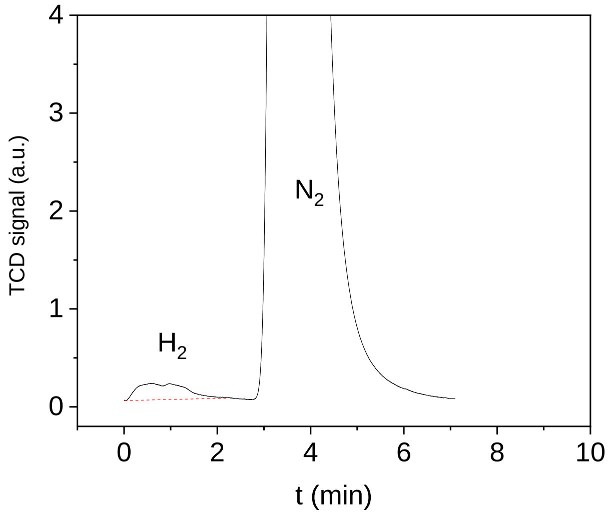

Fig. S13. **Detection of dihydrogen *via* gas chromatography.** Reaction conditions: THQ (1.25 mmol/L), mesitylene (8 mL), NC-800 (20 mg), room temperature, N_2 (1 bar), 5 d.

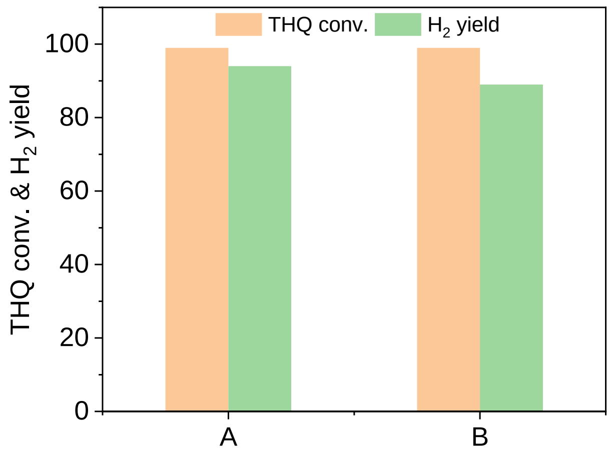

Fig. S14. **Quantification of H_2 production from THQ dehydrogenation at different reaction conditions.** Reaction condition A: THQ (12.5 mmol/L), mesitylene (8 mL), NC-800 (20 mg), 150 °C, N_2 (1 bar), 4 h. Reaction condition B: THQ (1.25 mmol/L), mesitylene (8 mL), NC-800 (20 mg), room temperature, N_2 (1 bar), 5 day.

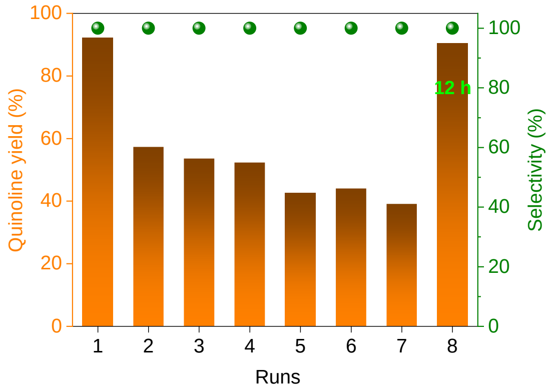

Fig. S15. Durability of NC-800 for metal-free THQ dehydrogenation. Reaction conditions: THQ (12.5 mmol/L), mesitylene (8 mL), NC-800 (20 mg), 150 °C, 3 h, N_2 (1 bar). The 8th recycling was performed at 12 h in order to achieve high yield of quinoline.

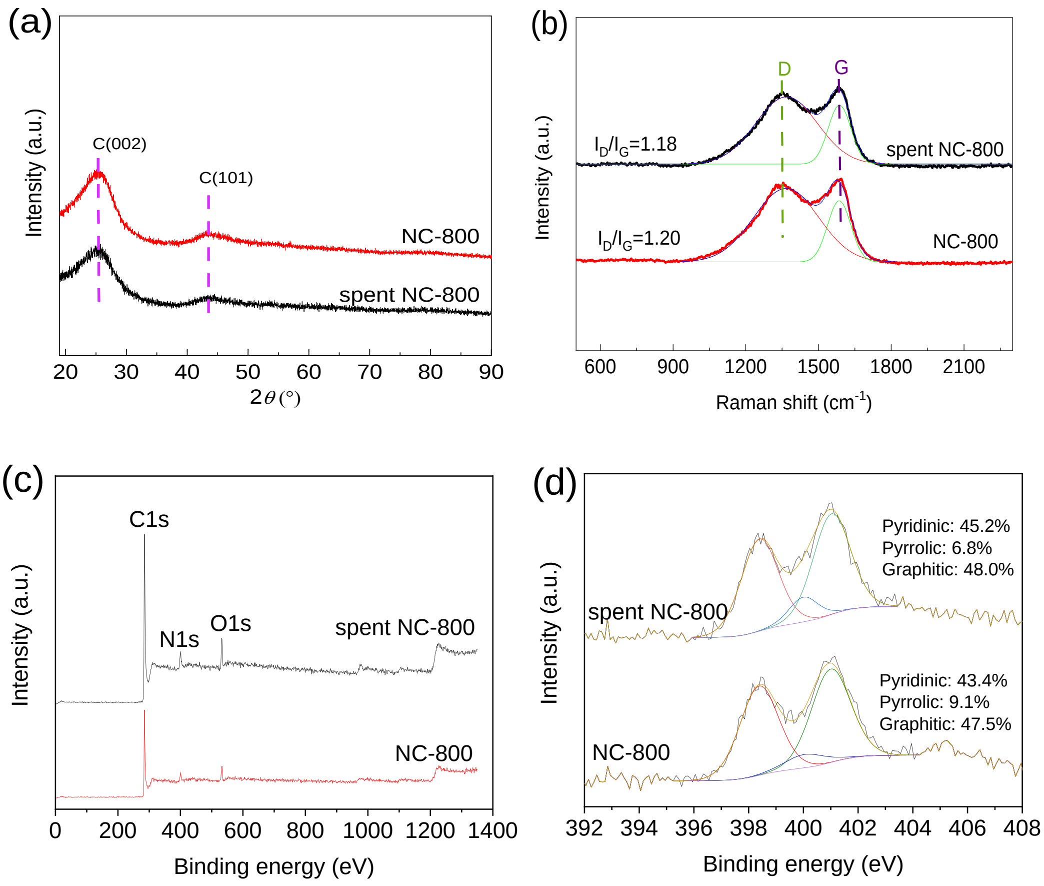

Fig. S16. Characterizations of fresh and recycled NC-800. (a) XRD patterns, (b) Raman spectra, (c) XPS wide-scan spectra and (d) N1s XPS spectra.

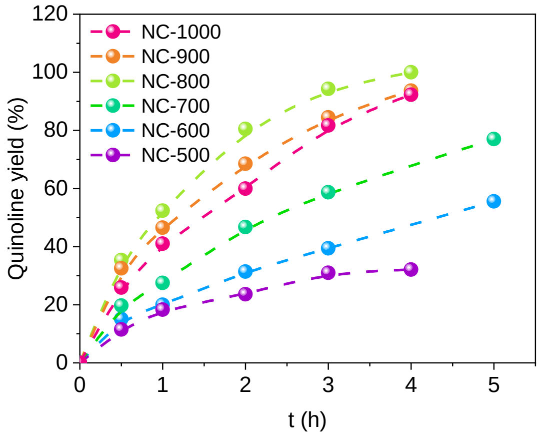

Fig. S17. Time courses of THQ dehydrogenation over various NCs. Reaction conditions: THQ (12.5 mmol/L), catalyst (20 mg), mesitylene (8 mL), 150 °C, 1 bar N_2.

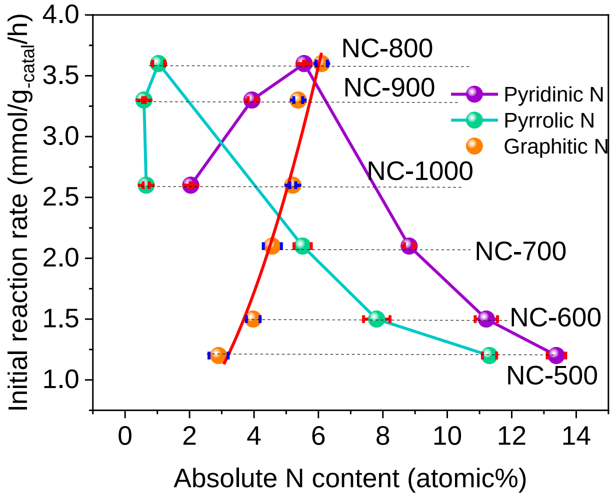

Fig. S18. Relationship between the reaction rate of THQ and different N species within the NC materials. No correlation of initial rates was found with N species except for graphitic N (Fig. 3a).

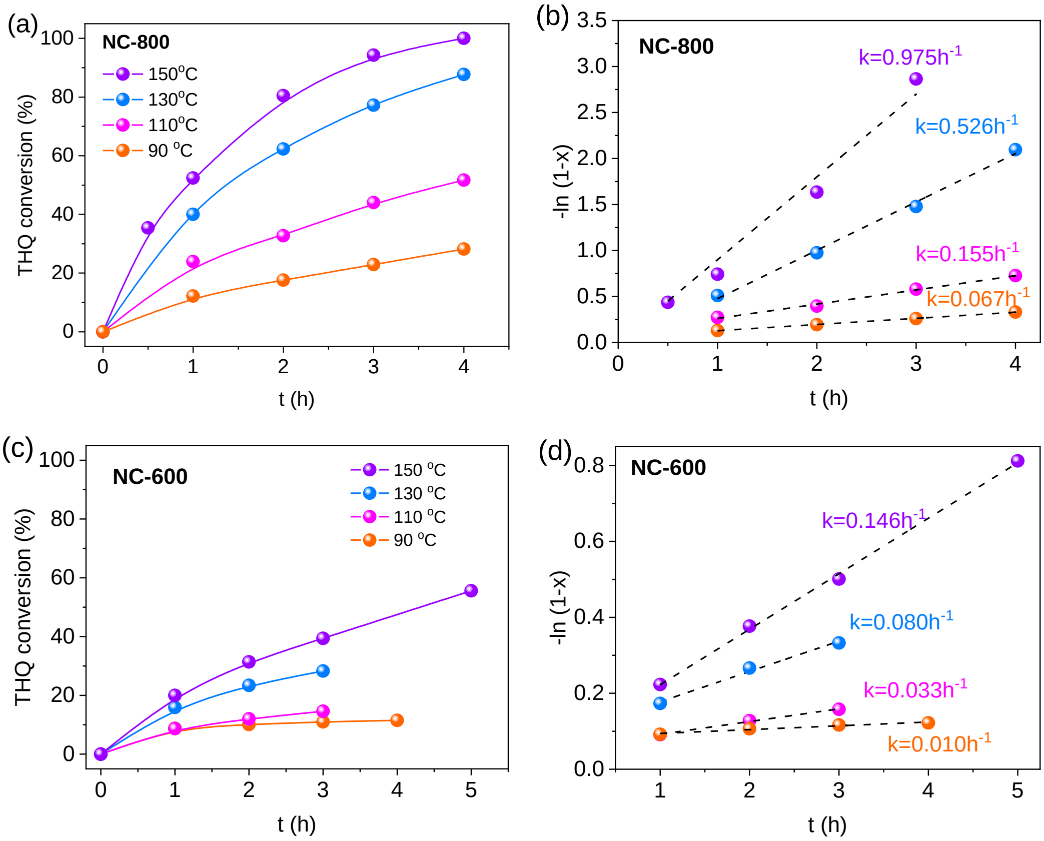

Fig. S19. Kinetic studies for dehydrogenation of THQ over NCs. (a and c) Time courses for dehydrogenation of THQ at different temperatures. (b and d) First-order kinetic fit for dehydrogenation of THQ. Reaction conditions: THQ (12.5 mmol/L), mesitylene (8 mL), catalyst (20 mg), N_2 (1 bar).

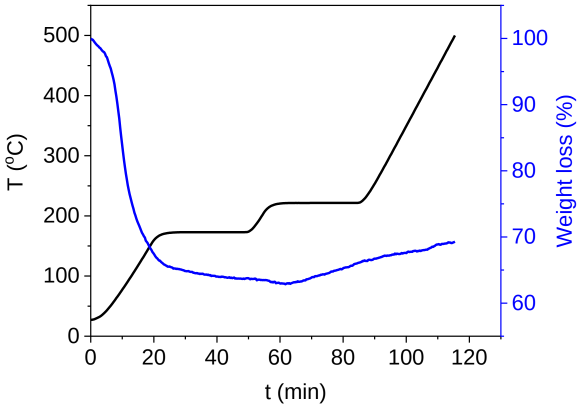

Fig. S20. TG-MS spectra of THQ-adsorbed NC-800.

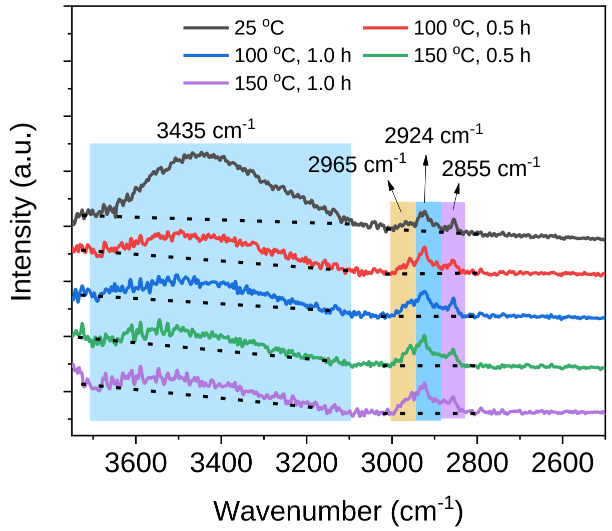

Fig. S21. ***In situ*** **DRIFTS of THQ adsorbed NC-800 at 25, 100 and 150 °C under flowing He.** As increasing temperature from 25 to 100 and 150 °C or prolonging treatment time to 0.5 and 1.0 h, N–H stretching vibration weakened remarkably, speculating THQ was gradually dehydrogenated into quinoline on NC-800. Meanwhile, the signals located at high wavenumbers (e.g., 2965 and 2924 cm^{-1}) other than low wavenumber (2855 cm^{-1}) were strengthened, which can be ascribed to the gradual aromatization of N-heterocycle of THQ.

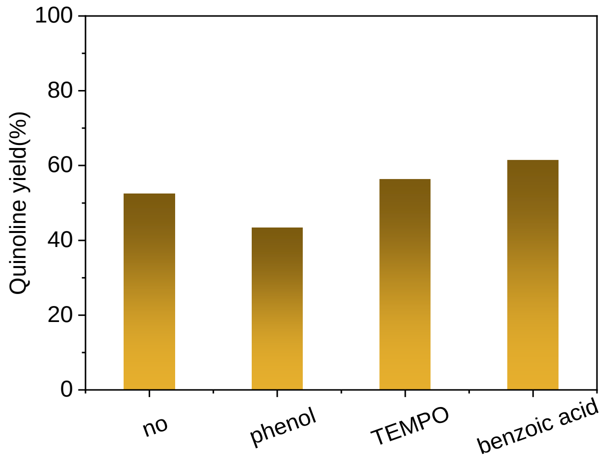

Fig. S22. Influence of additive on THQ dehydrogenation over NC-800. Reaction conditions: THQ (12.5 mmol/L), mesitylene (8 mL), additive (12.5 mmol/L), NC-800 (20 mg), N_2 (1 bar), 150 °C, 1 h.

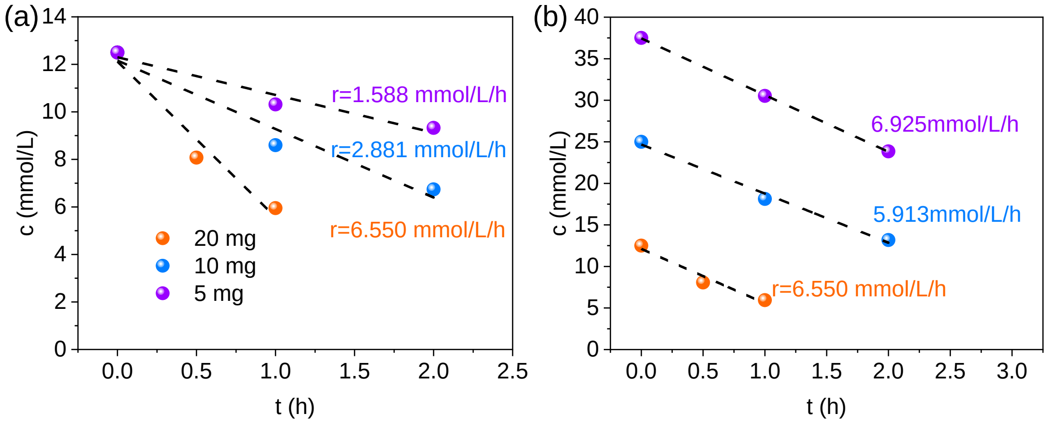

Fig. S23. Measurement of rate orders for catalyst dosage and THQ concentration at early reaction stage. (a) Influence of catalyst loading on THQ dehydrogenation. Reaction conditions: THQ (12.5 mmol/L), mesitylene (8 mL), NC-800, 150 °C, N_2 (1 bar). (b) Influence of substrate amount on dehydrogenation performance of NC-800. Reaction conditions: THQ, mesitylene (8 mL), NC-800 (20 mg), 150 °C, N_2 (1 bar).

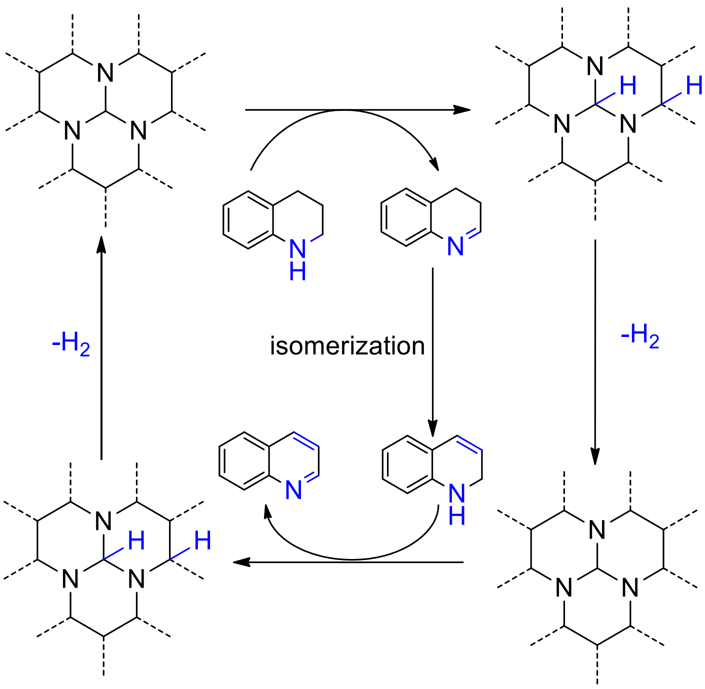

Fig. S24. Proposed mechanism of dehydrogenation of THQ over NC-800 based on DFT calculation. The structure of active sites was drawn according to our previous work,(*27*) in which closely-placed graphitic nitrogens (CGN) were active for activation of H_2 molecules. The CGN primarily originates from the nitrogen assemblies introduced by the diamine precursors into the graphitic carbons, which also enables the non-oxidative dehydrogenation of ethylbenzene and THQ and the selective hydrogenation of unsaturated functional groups in aromatics.

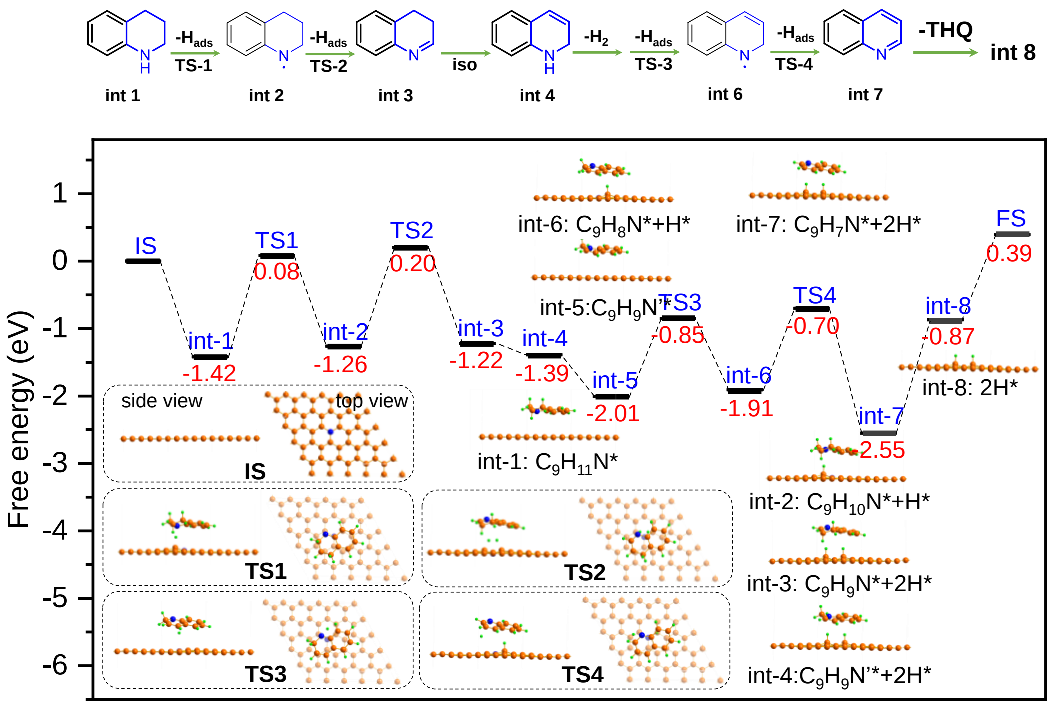

Fig. S25. Potential-free energy diagrams for dehydrogenation of THQ on isolated graphitic N (IGN) surface. It is well-known that graphitic N species are commonly existed in conventional N-doped carbon materials, but they are separated by surrounding carbon atoms and existed as isolated structure, especially at low N content within carbon materials. The label IS represents the initial state and the subsequent labels int1–int7 represent a series of intermediate states. The labels TS1–TS4 (TS is short for transition states) represent a series of transition states. The orange, blue, green spheres represent the C, N and H atoms, respectively.

Table S1. Pore parameters of NCs.

Catalyst	S_{BET} (m^2/g)	Pore volume (cm^3/g)	Pore size (nm)
NC-500	311	0.930	13.4
NC-600	345	1.196	13.9
NC-700	377	1.800	18.5
NC-800	334	1.244	14.7
NC-900	386	1.257	12.9
NC-1000	379	1.381	15.2

Table S2. Chemical compositions of NCs detected by XPS analysis.

Catalysts	Atomic concentration (%)		
	C	N	O
NC-500	66.2	27.6	6.3
NC-600	67.9	23.0	9.1
NC-700	72.2	18.9	8.9
NC-800	78.5	12.7	8.7
NC-900	81.6	9.9	8.5
NC-1000	88.0	7.9	4.1
Spent NC-800	80.1	13.8	6.0

Table S3. Relative content of N species based on N1s fitting data of NCs.

entry	catalysts	pyridinic N	error	pyrrolic N	error	graphitic N	error
1	NC-500	48.51	±0.93	40.98	±0.71	10.52	±0.96
2	NC-600	48.75	±1.35	33.96	±1.60	17.29	±0.83
3	NC-700	46.66	±0.69	29.16	±1.26	24.19	±1.33
4	NC-800	43.77	±0.16	8.20	±1.30	48.06	±1.22
5	NC-900	39.78	±1.06	5.91	±0.39	54.29	±1.25
6	NC-1000	25.82	±0.34	8.29	±1.07	65.90	±1.02

Table S4. Comparison of dehydrogenation of THQ in reported literatures.

Catalyst	Solvent	Atmosphere	Additive	*T* (º C)	*t* (h)	Yield (%)	Sele. (%)	TOF (h^{-1})	Ref.
NC-800	mesitylene	N_2	-	150	4	>99	>99	-	This work
NC-800	mesitylene	N_2	-	25	120	>99	>99	-	This work
Pd_3Au_1/CNT	xylene	Ar	-	140	12	96	96	26.8	(*9*)
Rh/TiO_2	*i*-PrOH	Ar	-	LED, room temperature	18	-	99	22.7	(*36*)
Co@NGS-800	CH_3OH	0.1MPa O_2	K_2CO_3	80	6	88	98	0.36	(*35*)
h-BCN	*i*-PrOH	Ar	-	LED, room temperature	12	86	86	-	(*37*)
Fe-L1@EGO-900	mesitylene	Ar	t-BuOK	145	24	88	90	0.46	(*13*)
PdNPs/SBA-15		Ar	-	130	23	99	99	0.92	(*38*)
Ni_2Mn-LDH	mesitylene	0.1MPa O_2	-	100	12	86	86	43.0	(*34*)
Pd_1Ni_4@MIL-100(Fe)	o-dichlorobenzene	Ar	-	110	12	100	100	27.1	(*39*)
FeO_x@NGr-C	heptane	1 MPa air	-	100	12	85	85	472.0	(*23*)
ISAS-Co/OPNC	mesitylene	Ar	-	120	8	99	99	9.83	(*11*)

Table S5. Metal-free transfer hydrogenation of quinolines over NC-800. Reaction conditions: substrate (20 mmol/L), FA (5 mmol), mesitylene (5 mL), NC-800 (20 mg), 180 °C, N_2 (10 bar).

Entry	substrate	product	t (h)	Conv. (%)	Selec. (%)
1	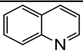	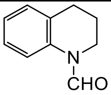	23	100	>99
2	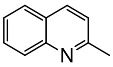	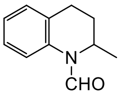	23	100	>99
3	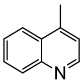	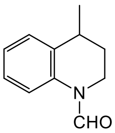	23	100	>99
4	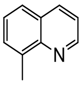	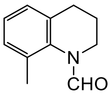	23	100	>99
5	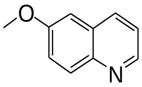	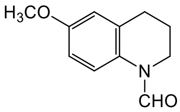	23	100	>99
6	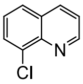	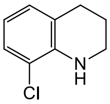	23	100	>99
7	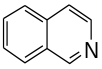	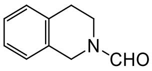	40	100	>99
8	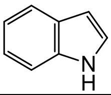	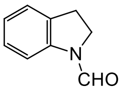	34	100	>99

Table S6. Absolute content of N species based on N1s fitting data of NCs.

entry	catalysts	pyridinic N	error	pyrrolic N	error	graphitic N	error
1	NC-500	13.39	±0.29	11.31	±0.22	2.90	±0.30
2	NC-600	11.21	±0.35	7.81	±0.41	3.98	±0.21
3	NC-700	8.82	±0.15	5.51	±0.27	4.57	±0.28
4	NC-800	5.56	±0.02	1.04	±0.19	6.10	±0.17
5	NC-900	3.94	±0.12	0.59	±0.04	5.37	±0.14
6	NC-1000	2.04	±0.03	0.65	±0.09	5.21	±0.09
